# Chemical Homogenization for Nonmixing Reactive Interfaces in Porous Media

**DOI:** 10.1021/acsomega.5c00641

**Published:** 2025-05-21

**Authors:** Winston Lindqwister, Manolis Veveakis, Martin Lesueur

**Affiliations:** † Faculty of Civil Engineering and Geosciences, 2860 Delft University of Technology, Stevinweg 1, 2628CN Delft, Netherlands; ‡ Department of Civil and Environmental Engineering, 3065 Duke University, 121 Hudson Hall, Campus Box 90287, Durham, North Carolina 27708, United States

## Abstract

Through rocks and concrete, batteries, and bone, porous media represent a wide class of materials whose chemical makeup and reactivity directly impact their behavior at multiple scales. While various theoretical and computational models have been implemented to capture the chemical behavior of these systems, none have investigated how the very geometry of porous media, the structures that make these materials porous and define the interfaces between solids and fluids, affects these behaviors. Through this work, we explored Minkowski functionals–geometric morphometers that describe the spatial and topological features of a convex space–to investigate how microstructural morphology affects systemic chemical performance. Using a novel asynchronous cellular automaton known as a surface chemical reaction network (CRN) to model chemical behavior, linkages were found between Minkowski functionals and equilibrium constant, as well as properties related to the dynamics of the microstructure’s reaction quotient. These quantities, in turn, give insight into how morphology affects bulk porous media properties, such as Gibbs’ free energy.

## Graphical abstract

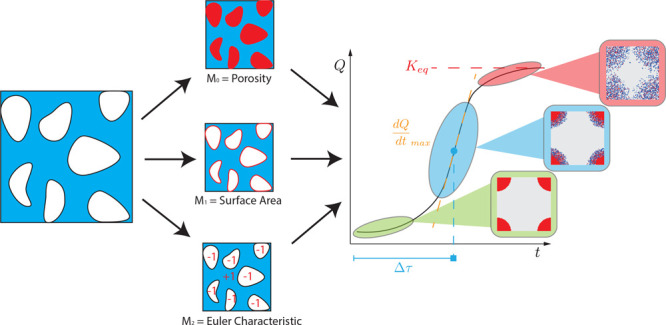

## Introduction

While ubiquitous, from bone to rock to fuel cells, porous media represent a wide class of materials that remain challenging to fully characterize in terms of multiscale effects. Their properties at the microstructural level have been shown to be intrinsically linked to mesoscale behavior, yet the exact nature of this scaling has proven to be highly elusive due to the complicated nature of modeling multiscale phenomena.^1,2^ In order to link these effects, one approach has been to use geometric morphometers as a basis for deriving energetic relationships from microstructural form to porous media behavior.^3–5^ A class of geometric morphometers of particular interest is Minkowski functionals, which have been shown to be powerful descriptors as a basis for linking form to function in many important properties of porous media, from resistivity to permeability.^6–8^

Minkowski functionals are geometric morphometers, characterizing both morphology and topology of spatial patterns, that are conceptualized from the field of statistical physics.^9^ They have seen wide application in describing phenomena from the spin of galaxies^10^ to the permeability of porous media.^6^ The use of these functionals as a descriptor for mesoscale systems is supported by Hadwiger’s theorem,^11^ which guarantees that for a polyconvex, isotropic body of dimension *D*, *D* + 1 Minkowski functionals can be used to sufficiently describe the behavior of the system. In particular, Minkowski functionals have been shown to have a powerful connection between geometry and free energy, creating an important linkage between structural and energetic properties of materials.^11^

One property of porous media that is of particular interest, yet is notoriously challenging to link to multiple scales, is the quantification of chemical behavior.^12–15^ Chemical activity in porous media drives both immediate behavior^16,17^ and long-term performance,^18–20^ and is an important factor in modeling pollutant transport,^21,22^ flows of nutrients in cells,^23,24^ and carbon sequestration.^25,26^ Unfortunately, while the need for understanding chemical behavior in porous media is essential, the means to do so are heavily complicated due to porous media’s inherent interfacial nature, leading to divergence from classical, well-mixed models.^27,28^ In the world of modeling well-mixed systems, the classic approach to homogenization is through chemical reaction networks.^29,30^ Chemical reaction networks (CRNs) are graph-based models of dynamic chemical interactions that typically organize chemical species as functions *f*(*x*) and their evolution *ẋ* to form a continuous autonomous dynamic system of the form *ẋ* = *f*(*x*). These models provide powerful tools in identifying reaction steady states,^31^ steady state stability,^32^ persistence,^33^ existence of stable periodic solutions,^34^ and performing model reduction.^35,36^

While these models are quite powerful in driving the understanding of these complex dynamic systems, there are certain assumptions of a traditional CRN that limit their ability to fully characterize interfacial complexes such as porous media. Namely, CRN models typically assume a well-mixed arrangement of comparable density across the entire domain.^37^ To address this limitation, Qian and Winfree proposed a novel method for implementing a CRN on a surface, applying a graph structure to a geometric boundary with CRN-like kinetics.^38^ This method, known as a surface CRN, is implemented as an asynchronous cellular automaton with probabilistic transition rules that mimic a continuous-time Markov chain process. Through Qian and Winfree’s work, as well as advancements from Clamons et al., surface CRNs have demonstrated the ability to form dynamic spatial patterns, operate as DNA circuits, and model adsorption and desorption behavior on a surface.^38,39^ Through this work, we extend the implementation of these models to solid–fluid interfacial behavior on a porous microstructure, with a linkage back to Minkowski functionals for a succinct characterization of macroscale microstructural performance via microscale properties.

## Methods

### Surface CRNs

In order to model chemical behavior, surface CRNs were selected as the simulation medium. A surface CRN resembles the rules of a classic CRN modeling approach but crucially imposes spatial constraints on the manner in which reactions can occur. By definition, a surface CRN is an asynchronous, stochastic cellular automaton with CRN-like transition rules.^38^ Informally, this can be seen as a CRN where individual chemical species are localized to sites on a specific surface and may only interact with neighboring molecules.^39^ On a technical level, a surface CRN is a continuous-time Markov chain defined by a lattice *L* of connected sites *i* ∈ *L* with each site defined by a state *s*_*i*_ and each site defined as *i*. The ability to switch states is determined by a set of unimolecular or bimolecular transition rules *r* ∈ *R*, where each reaction is defined as *A* → *B* or *A* + *B* → *C* + *D*, with the rate of each reaction as λ_r_. As an asynchronous cellular automaton, each reaction occurs independently, with the ordering of these reactions processed via a queuing system. Essentially, at each frame of the simulation, the simulation grid is queried for all potential reactions that may occur based on each node’s neighbors, and each potential reaction has the time for its occurrence drawn from an exponential distribution. This *time to next reaction* Δ*t* is calculated as follows:

$$\Delta t=\log\left(\frac{1}{\mathrm{rand}\left(x\right)}\right)\left(\frac{1}{\lambda_{r}}\right)$$ 1

with rand­(*x*) serving as a random draw from a uniform distribution bounded between 0 and 1. After each *time to next reaction* is calculated for all candidate nodes, each node has its corresponding reaction scheduled for time *t* + Δ*t* and pushed to a priority heap queue. From here, the first reaction from the queue is popped and processed, changing the respective reactants to products. With the new map in place, the current time of the simulation is set to *t* = *t* + Δ*t*, and all reactions in the queue involving sites changed in the aforementioned step are removed. The new site species are checked for any potential reactions, and these are added to the queue as previously described, and this is repeated until a stop condition is met. Simplified, this can be seen as

1. Initialize with a global state grid at time *t* = 0.
2. Scan each node for potential reactions that can occur, calculate the *time to the next reaction t* + Δ*t*, and add it to a priority heap queue.
3. Pop the first reaction in the queue and process reactants to products, setting the new time as *t* = *t* + Δ*t*.
4. Remove all reactions involving the same sites as the current reaction site in question from the queue.
5. Scan the products in the current site for new potential reactions, and recalculate and add to the queue as in step 2.
6. Continue from step 3 until a stop condition (such as the maximum duration of the simulation being reached or an empty queue) has been met.

As described in Clamons et al.,^39^ the total time complexity of the simulation is *O*(*n* + *r*log *w*), where *n* is the number of sites in the surface or the CRN, *r* is the total number of reaction events simulated, and *w* is the maximum number of reactions in the queue at any given time.^39^

Although surface CRN reactions may only take transition rules as chemical reactions, other surface/species behavior may be emulated using the relative flexibility of what is defined as a “reaction”. For example, by default, surface CRNs do not allow for the diffusion of molecules. However, in this work, diffusion of molecules is simulated using reactions of the form $X+E\overset{k}{\to }E+X$ , where *X* is the diffusing species in question, *E* represents an exmpy site that said species can travel to, and *k* controls the rate of diffusion.

While qualitative in nature, surface CRNs provide a simple and straightforward model of CRN-like chemistry that accounts for the geometric considerations of an interface-sensitive chemical system that a typical CRN model cannot provide. Compared to other discrete stochastic reaction-diffusion models, such as Kinetic Monte Carlo (KMC) and stochastic reaction-diffusion simulations, surface CRNs come with a host of advantages and trade-offs. The primary difference between surface CRNs and other models is the requirement for species to exist in discrete spaces compared to continuous positions of species.^40^ This allows surface CRNs to naturally capture macromolecular crowding behavior, as well as to preserve the local geometry of chemical reactions.^41^ The relative simplicity of calculating surface CRN switching rules also makes them highly parallelizable; every reaction occurs in a queue and is processed one-at-a-time. One could easily segment a space into multiple surface CRNs, allowing for rapid parallel processing of large-system behavior.

For this study, a dissolution reaction was studied to understand the linkage between Minkowski functionals and, by extension, microstructural geometry and chemical behavior. The dissolution reaction is of the form:

A+B⇌2P 2

with *A* defined as a reactive solid species, *B* as a reactive liquid, and *P* as a reaction liquid product. This reaction is a generic form of a reversible fluid-release reaction where no solids are produced in the forward reaction and the liquid products are not mixed with preexisting fluids. This makes the transition rules of the reaction at a solid–liquid interface straightforward, since no solid is retained. Indeed, this is reflected in Table 1 which lists the input transition rules for the surface CRN simulator. It is to be noted that this choice of interface reaction is constraining the conclusions of the present study to nonmixing fluid-release reactions rather than to any generic interfacial reaction. This class of reaction resembles the behavior of any solid dissolving readily in an environment of excess fluid.

**Table 1:** Transition Rules for the Benchmark Diffusion Reaction

transition rule	reaction rate
*A* + *B* → *P* + *P*	0.4
*P* + *P* → *A* + *B*	0.1
*P* + *B* → *B* + *P*	1.0

### Minkowski Functionals

With a means to simulate chemical behavior defined, the linkage of these chemical results to microstructural morphology must be quantified. Minkowski functionals are geometric and topological descriptors derived from integral geometry used to describe spatial patterns.^42^ For a domain of dimension *D*, *D* + 1 functionals are required to describe it. In the case of a 2D body with a surface Ω and a smooth boundary δΩ, the required functionals are defined as

$$\begin{array}{rll} M_{0}(\Omega ) & =\int_{\delta \Omega }dA & \\ M_{1}(\Omega ) & =\frac{1}{2}\int_{\delta \Omega }dL & \\ M_{2}(\Omega ) & =\frac{1}{2}\int_{\delta \Omega }k(\Omega )dL=\pi \chi & \end{array}$$ 3

where d*A* is defined as a surface element, d*L* is a line element, and *k*(Ω) is the signed curvature. For our 2D system, *M*_0_ corresponds to the surface area of the porous domain, *M*_1_ as the perimeter, and *M*_2_ as the signed curvature, which is directly proportional to the Euler characteristic χ via the Gauss–Bonnett theorem.^5^ For any functional ***M*** (Ω) that is additive, motion-invariant, and continuous, per Hadwiger’s theorem,^11^ this functional can be described as a linear combination of Minkowski functionals *M*_*n*_(Ω) as follows:

$$M(\Omega )=\sum_{n=0}^{d}c_{n}M_{n}(\Omega )$$ 4

### *K*_eq_ Selection

In order to study the effect microstructural morphology characterized by Minkowski functionals has on chemical reaction behavior, a simple dissolution reaction, as described in eq 2 was studied. The global chemical response is classically characterized through a measure of the total extent of the reaction determined by the reaction quotient *Q*_r_ and its value at steady-state called the equilibrium constant *K*_eq_ for nonmixing systems.

These descriptors will form the basis of a relationship between Minkowski functionals and the change in Gibbs free energy Δ*G*, as seen in the thermodynamic relation:

$$\Delta G=RT\ln\;\frac{Q_{r}}{K_{\mathrm{eq}}}$$ 5

with *R* as the universal gas constant and *T* being the temperature. This quantity not only gives information as to what direction a reversible reaction occurs but also plays into the maximum work evolved from thermodynamic processes in a system. An important quantity related to the change in Gibbs free energy of a system is the change in the standard free energy of a system (Δ*G* ^0^), defined by the relation:

$$\Delta G^{0}=-RT\ln\;K_{\mathrm{eq}}$$ 6

Δ*G* and Δ*G* ^0^ are linked via the expression

$$\Delta G=\Delta G^{0}+RT\ln\;Q_{r}$$ 7

Δ*G* ^0^ is the change in Gibbs free energy of a substance at 1 bar of pressure and a temperature of 25 °C.

In classical mixing systems, the forms of *K*_eq_ and *Q*_r_ for the dissolution reaction described in eq 2 are derived from the law of mass action based on the reaction coefficients of each species, as follows:

$$\begin{array}{rll} K_{\mathrm{eq}} & =\frac{[P_{\mathrm{eq}}{]}^{2}}{\left[A_{\mathrm{eq}}][B_{\mathrm{eq}}\right]} & \\ Q_{r} & =\frac{[P{]}^{2}}{\left[A][B\right]} & \end{array}$$ 8

Equilibrium values used to calculate *K*_eq_ take the mean of the last few values of the species concentrations at steady state, reducing the overall noise for the calculations. In order to meaningfully calculate the entire *Q* evolution without overpropagation of noise, a Whittaker–Eilers filter was applied, as detailed in Appendix I.

From the work of Boelens and Tchelepi,^42^ an agreement between Minkowski functionals and the change in Gibbs free energy of the following form is expected based on additive concepts of thermodynamics:

$$\Delta G=RT\ln\;\frac{Q}{K_{\mathrm{eq}}}=\alpha M_{0}+\beta M_{1}+\gamma M_{2}$$ 9

Based on this relation, a linear combination of Minkowski functionals in an exponential distribution would describe the dynamic energetics of the bulk microstructure.

However, nonmixing systems have been shown for over 50 years to deviate from the law of mass action.^43^ The burgeoning work of surface chemistry energetics has added a new perspective on these considerations, suggesting that the traditional law of mass action described in eq 8 is not accurate in systems with multiple state phases.^44–46^ Bauermann et al.^44^ instead define *K*_eq_ as a relationship between stoichiometric coefficients, activity coefficients, and reference chemical potentials, suggesting slower versions of *K*_eq_ for nonmixing interface reactions based on these metrics. Unfortunately, in this synthetic dissolution reaction, these considerations are not readily applicable since energetic terms like chemical potential and activity coefficients are assigned *a priori* in the form of transition rule rates and diffusion rates, respectively. As a result, we can only homogenize numerically, and thus, three *K*_eq_ formulations will be tested, those from eq 8 and two slower versions defined as

$$K_{\mathrm{eq}}^{R^{n}}=\frac{[R_{\mathrm{eq}}{]}^{n}}{\left[Q_{\mathrm{eq}}][A_{\mathrm{eq}}\right]},n=1;3/2;2$$ 10

The results from these varying *K*_eq_^*R* ^*n*^^ calculations will inform an ultimate selection for the *K*_eq_ criteria on which Minkowski functional analysis will be based. Further to the extent of the reaction, its equivalent rate will be represented through a characteristic time of the reaction to reach its maximum rate, $\frac{dQ}{dt_{\max}}$ , through the value Δτ. These will both, in turn, be investigated as a function of Minkowski functionals of an assumed form:

$$\frac{dQ}{dt_{\max}}=f(c_{n}M_{n})$$ 11

and

$$\Delta \tau =f(c_{n}M_{n})$$ 12

These quantities and how they are represented in the sCRN simulation can be viewed in Figure 1.

**Figure 1:**
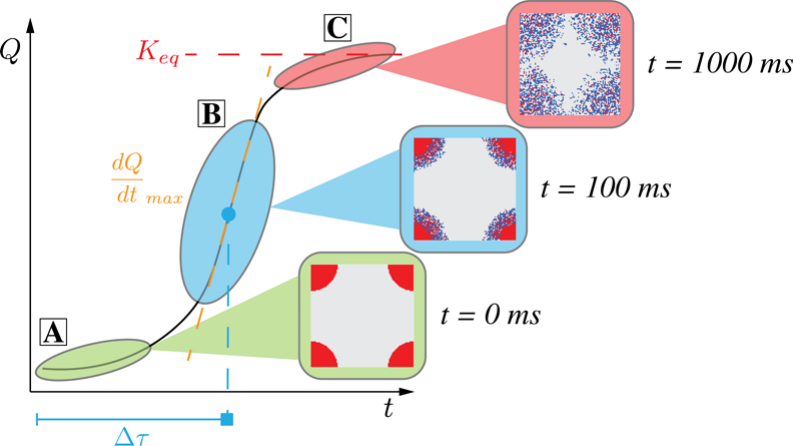
Summary diagram of the quantities measured and how they are mapped to a typical sCRN simulation for dissolution. (A) Initiation phase of the reaction, with almost no product created. (B) Point of the reaction with the greatest amount of reactant being produced at any given moment, denoted by ${\frac{dQ}{dt}}_{\max}$ , and the time to reach this point as Δτ. (C) Reaction eventually reaching equilibrium, or *K*_eq_.

### Surface CRN Qualitative Validity

Surface CRNs are designed to represent chemical behavior. The rules on reaction rates mirror actual chemical reaction forward and backward rates, and the inherent spatial dependence of the system is representative of real-life chemical systems that are contact- and interface-dependent. The expected analytic behavior of the dissolution reaction described in Table 1 and eq 2 is expressed through the following system of equations:

$$\begin{array}{rll} \frac{dA}{dt} & =2k_{r}P-k_{f}AB & \\ \frac{dB}{dt} & =2k_{r}P-k_{f}AB & \\ \frac{dC}{dt} & =k_{f}AB-2k_{r}P & \end{array}$$ 13

with *k*_r_ and *k*_f_ representing the forward and backward rates of reaction. Solved analytically and compared to the surface CRN results seen in Figure 2, we see that qualitatively, the surface CRN chemical evolution follows the expected behavior, albeit with differing time and concentration scalings. This further matches the behavior expected of reaction concentration evolution found in the analytical chemistry literature.

**Figure 2:**
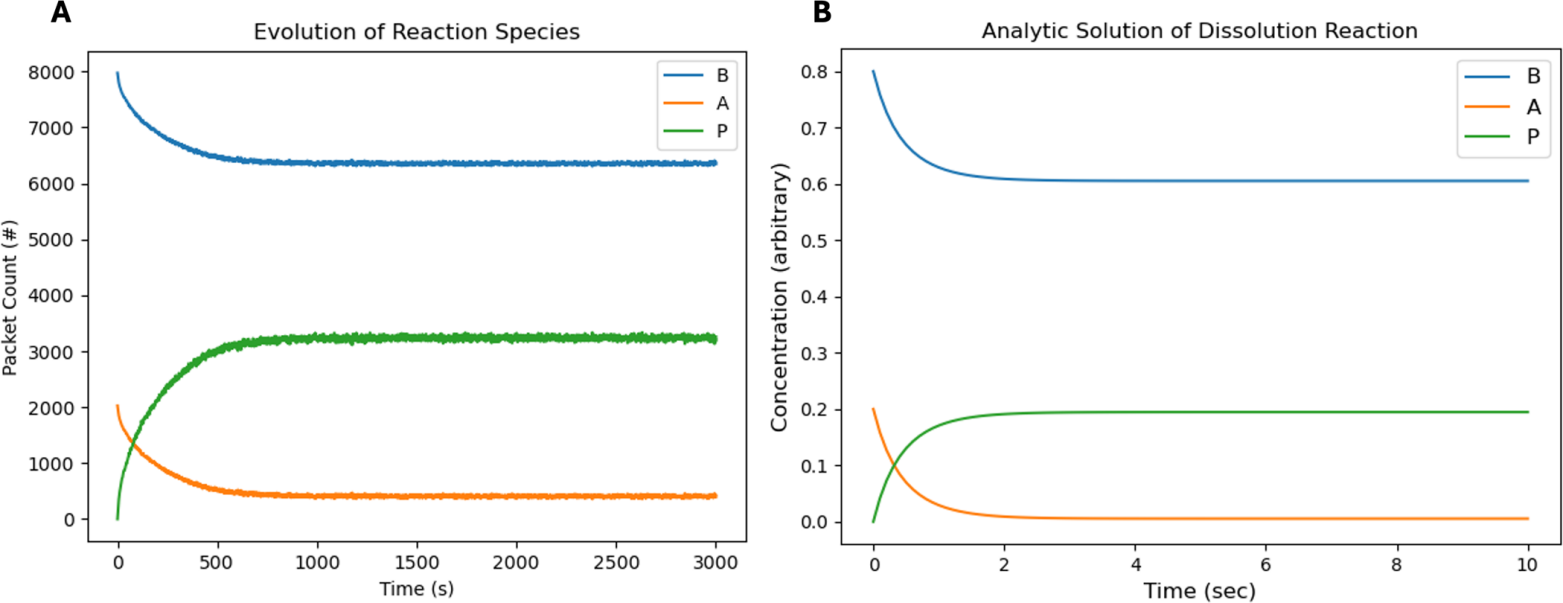
Surface CRN chemical species evolution vs the analytic solution of the system of chemical equations found in eq 13. (A) Computational solution via surface CRNs, while (B) represents the analytic solution solved.

## Simulations on Synthetic Microstructures

### Microstructure Selection

While real-world porous microstructures are highly stochastic with vastly complicated pore networks, separating the effects of individual microstructural morphological features is highly challenging due to the inherent interconnected nature of Minkowski functionals. For example, it is incredibly difficult to take a fully stochastic microstructure and vary its porosity without also changing its surface area and Euler characteristic. Indeed, while these functionals are by definition linearly independent, it is quite difficult to create a schema to generate microstructures that only vary one functional while fixing the others. To address this, we opted for a unit cell approach to approximate porous microstructure features, preserving the solid-void interplay of porous materials while keeping the geometry as controlled as possible for functional isolation. For that purpose, we designed three types of microstructures.

Figure 3 displays the first microstructural design, a periodic unit cell representing a close idealization of a porous granular material. Each unit cell is designed as an *N* × *N* pixels square with four circles of equal radius *r* at each corner. White pixels represent solid species, while black pixels represent voids for fluid species to diffuse. Each edge of the unit cell is a periodic boundary, allowing chemical reactions to occur from one end of the cell to the other. To generate unit cells of differing Minkowski functionals, the unit cell bounding box is fixed at side length *N* as *r* is varied. While eq 3 holds as the basis for calculating Minkowski functional values, *M*_0_ and *M*_1_ are nondimensionalized by the reference length *N* of the bounding box. Thus, Minkowski functionals are calculated as follows:

$$M_{0}=1-\frac{\pi r^{2}}{N^{2}}$$ 14

$$M_{1}=\frac{2\pi r}{N}$$ 15

$$M_{2}=V-E+F$$ 16

with *V*, *E*, and *F* of eq 16 representing the vertices, edges, and faces of the microstructure, respectively. Note that eq 14 is calculated as the porosity of the microstructure (fraction of void to the total box size). For unit cell tests, *M*_2_ is held constant (χ = 1 for a circle split into four slices) while *M*_0_ and *M*_1_ vary with *r*.

**Figure 3:**
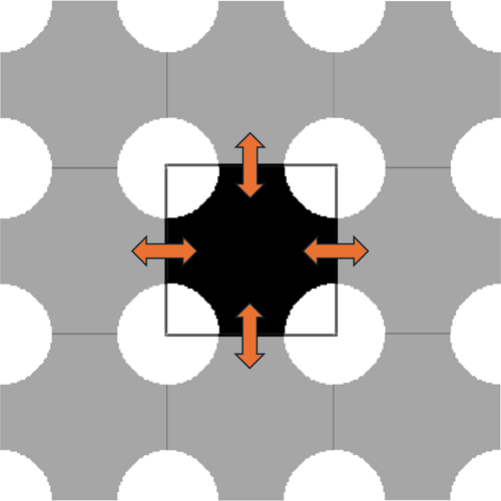
Example of a unit cell microstructure. The radius of the circles at the corners is varied per individual unit cell designs. The boundary of the unit cells is periodic, allowing for chemical reactions to occur from one edge to another.

In order to separate the effects of *M*_0_ and *M*_1_, a second test was designed to hold *M*_0_ and *M*_2_ constant while only varying *M*_1_. In Figure 4, an example of the microstructure is shown. The interface between the solid region and the void region of the microstructure has a periodic wave applied to it. The perimeter of the interface can be varied while keeping the same area ratio from the solid to fluid regions. The number of waves on the perimeter is denoted by the wavenumber ν. The perimeter and area of the wavy interface are calculated in a similar manner to that of an ellipse; thus, *a* and *b* represent shape measures for calculating wave area and perimeter. Because of the periodic nature of the wave, *M*_0_ and *M*_2_ remain constant while *a*, *b*, and ν are varied (assuming ν remains an even number). Based on the Ramanujan approximation for the perimeter of an ellipse, *M*_1_ is calculated as

$$\begin{array}{rll} M_{1} & =\frac{\nu \pi \left(a+b\right)}{2}\left(1+\frac{3h}{10+\sqrt{4-3h}}\right) & \\ h & =\frac{{\left(a-b\right)}^{2}}{{\left(a+b\right)}^{2}} \end{array}$$ 17

**Figure 4:**
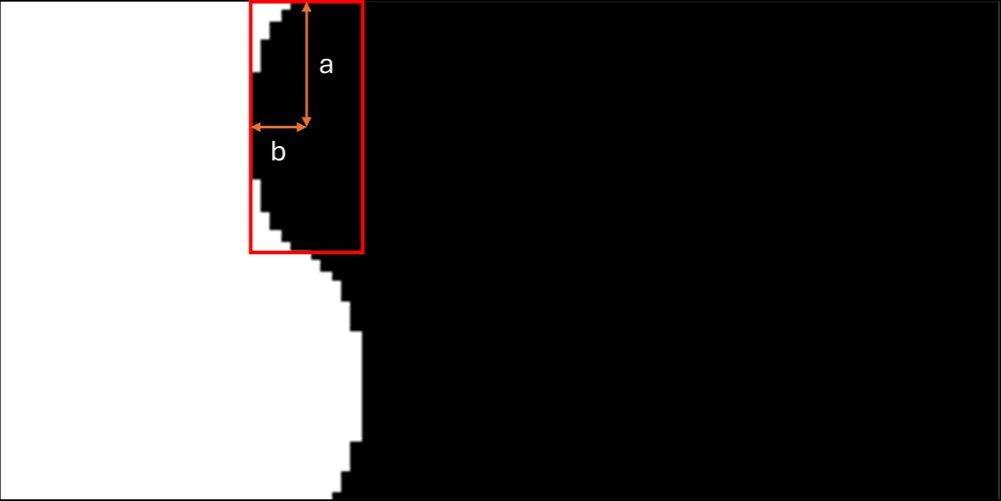
Example of a perimeter test microstructure. *a* and *b* control the wave properties along the perimeter, varying *M*_1_ while maintaining a constant *M*_0_. One periodic wave is highlighted in the red bounding box, with the wavenumber of the cell defined as ν.

The final microstructural design aims to maintain constant *M*_0_ and *M*_1_ while varying *M*_2_. This test matches the method used from ref 42 for varying Euler Characteristic while maintaining constant porosity and surface area. Figure 5 represents how this test was performed with a circle of solid material immersed in a bounding cell of fluid. As pixel-sized holes are added to the circle, χ, and therefore *M*_2_, decreases. Due to the small size of these holes, *M*_0_ and *M*_1_ change negligibly through the test.

**Figure 5:**
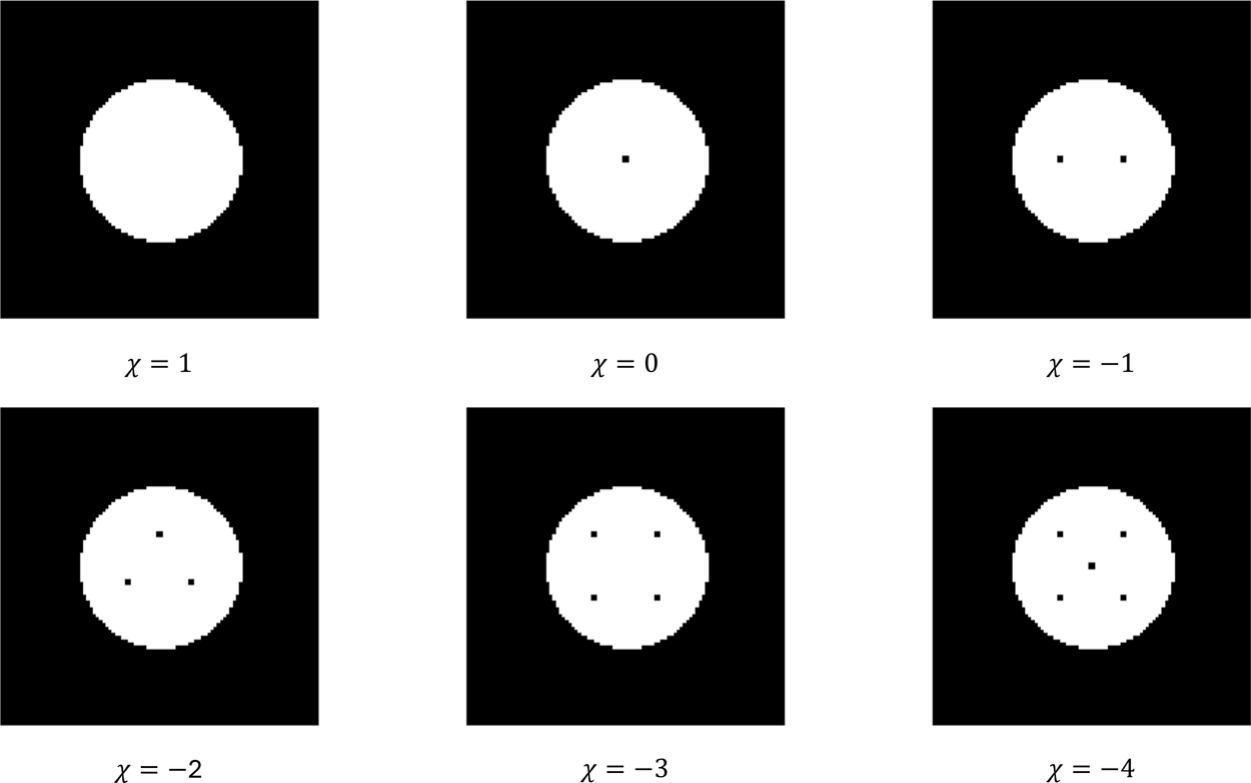
Euler characteristic test. All microstructures are circles of constant radius with pixel-sized holes added. Each hole lowers χ by 1, at a negligible change in porosity and perimeter.

### Resolution Convergence

In order to assess the validity of surface CRNs as a modeling tool for chemical behavior, a resolution convergence study is performed to verify that *K*_eq_ values scale directly with simulation resolution but converge to a stable value at high resolution.

For this resolution convergence study, repeated simulations were performed in the periodic unit cell, varying the side length of the cell *N* while keeping the ratio of side length to circle radius *r* in a consistent 4:1 ratio *N*:*r*. This ratio was selected because it is in the middle of the range of cell-to-circle ratios, allowing for maximum generalizability in the range of resolutions tested. At low porosities (about a 2:1 ratio), the unit cell is not sufficiently saturated with reactive fluid, considerably changing the surface CRN behavior. In essence, at this point of subsaturation, there are not enough nodes for the reactive fluid in the unit cell to fully dissolve the solid structure, making the reactivity of the material bottlenecked by the diffusivity of the surrounding fluid. We chose porosity ranges away from this effect to negate this diffusion bottleneck and thus chose a mesh convergence analysis point away from this limit. Due to increased resolution, dynamic effects in the unit cell would need to be scaled via the transition rule rate laws to remain consistent, as the increased resolution would effectively increase the “distance” each set of molecules would need to travel due to the fixed grid nature of surface CRN simulations. For this reason, we only compare steady-state solutions and look at the convergence of *K*_eq_^*R*^ with resolution.

As seen in Figure 6, *K*_eq_ values show a clear exponential decrease with increasing resolution, converging at a stable solution at about *N* = 200, and this is the resolution used for all simulations.

**Figure 6:**
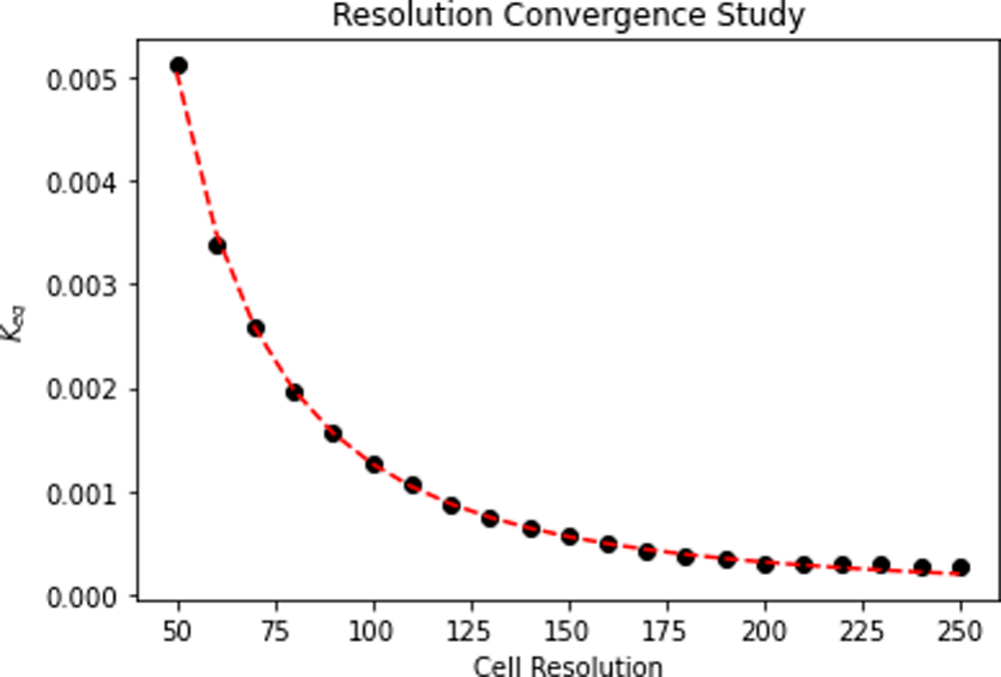
Resolution convergence study, varying unit box size.

### Rate Effects

According to the work of Boelens and Tchelepi,^42^ the primary discrepancy in *K*_eq_ values found in interfacial, nonmixed systems compared to well-mixed systems manifests from differing reaction rates, both within the separate phases but also in the transition from one phase to another. Essentially, the additional phase change adds an energetic hurdle for the dissolution reaction to occur, changing the overall reaction rate of the forward reaction and thus lowering the overall equilibrium coefficient. In surface CRN simulations, these discrepancies can manifest in the *a priori* transition rule rates, as well as the assigned diffusion rate for “mobile” species in the simulation space. The changing diffusion rate of the surface CRN mirrors this behavior to an extent. At higher diffusion rates, reacted products of the surface CRN reaction are much more likely to leave sites adjacent to nonreacted nodes, allowing for more chemical reactions to occur. This effectively increases the forward rate of the reaction, thus increasing the *K*_eq_ value of the system. Figure 7 demonstrates how an increasing diffusion rate increases *K*_eq_ consistently across varying methods for the *K*_eq_ calculation. All of these increases are closely matched to a power law, with consistent power scaling across all three calculation schemes. The primary difference in each curve comes from the order of magnitude of [*R*] at a consistent linear scaling.

**Figure 7:**
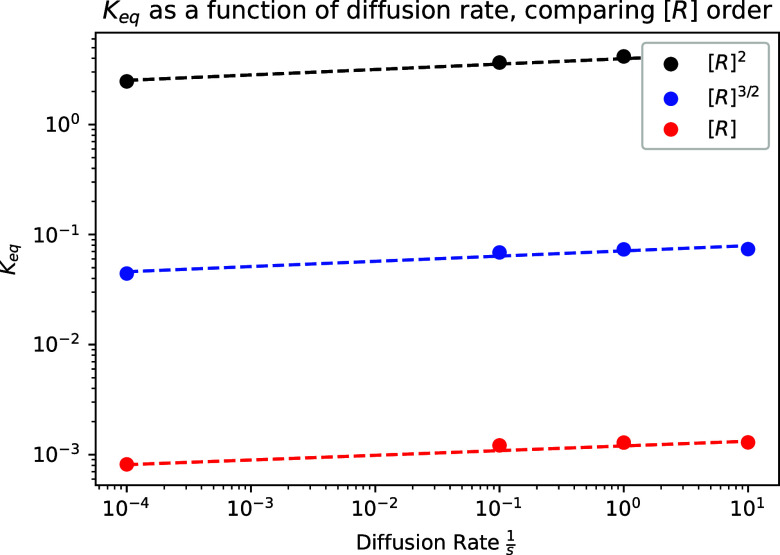
Effect of diffusion rate on the *K*_eq_ of the system for each *K*_eq_ formulation.

A similar study was conducted by comparing the reaction rate in transition rules. As detailed in eq 2, the diffusion reaction is a reversible reaction that in its initial form favors a forward reaction. For this study, the ratio of forward reaction *k*_f_ to reverse reaction *k*_r_ was varied, as shown in Figure 8. Similar to the behavior exhibited in Figure 7, *K*_eq_ calculations varied consistently across the same order of power law, modulating by constant orders of magnitude per the *K*_eq_ formulation.

**Figure 8:**
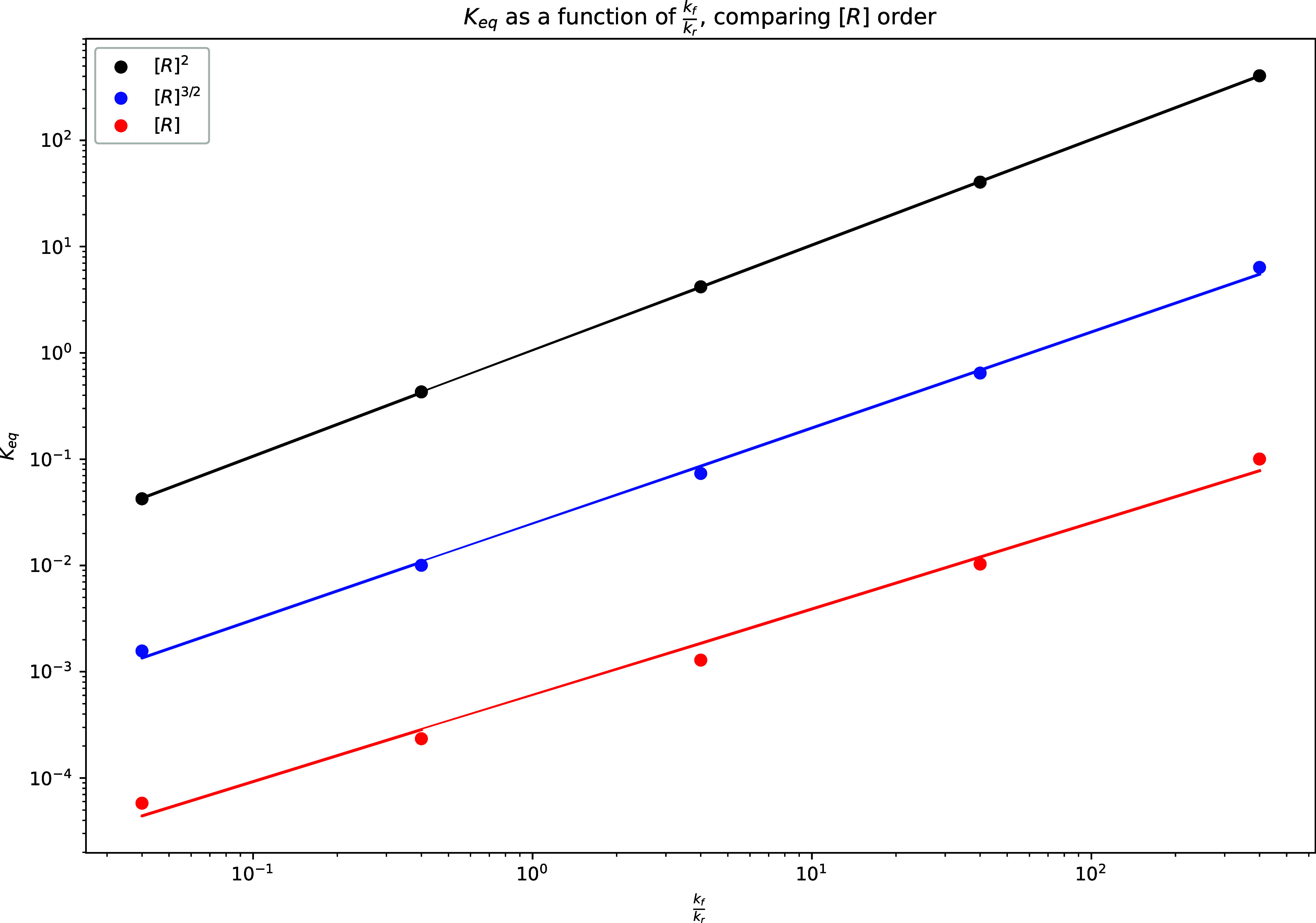
Effect on the reaction rate ratio for the forward and reverse reaction on *K*_eq_ for each *K*_eq_ formulation.

Both rate effect studies shared consistent results in terms of the scalability of *K*_eq_ calculations across various rate schemes and diffusion rules. The influence of these varying rates points to the validity of Boelens’ work, as the kinetics of the varying phases of the reaction, both chemically and physically, have a direct influence on the overall steady state behavior of the system.

## Results

### Effects of Microstructure Geometry

Through the course of this study, microstructural geometry and morphology had a visible effect on the chemical behavior of the porous microstructure. This is seen through the thermodynamic properties of *K*_eq_, as well as the quantities $\frac{dQ}{dt_{\max}}$ and Δ. Results relevant to the main conclusions of this manuscript are discussed in the following sections, while the full set of results relevant to each Minkowski functional and each thermodynamic quantity can be found in the article’s Supporting Information.

### Unit Cell

As discussed above, when investigating the effects of Minkowski functionals on the chemical properties of the system, a clear definition of *K*_eq_ must be selected. In Figure 9, it is clear that depending on the selected scheme of calculating *K*_eq_, as highlighted in eq 10, the reference scaling and relational behavior with regard to radius changes dramatically. Figure 9B shows a weak linear, bordering on trivial, relationship between radius and *K*_eq_^*R*^. Figure 9C, on the other hand, shows a strong linear relationship between radius and *K*_eq_^*R* ^3/2^^. Finally, Figure 9D shows a strong exponential relationship between the radius and *K*_eq_^*R*^. Note that the differentiation in these schemes only appears in the calculation of *K*_eq_ itself, but not in other kinetics-related factors such as Δτ.

**Figure 9:**
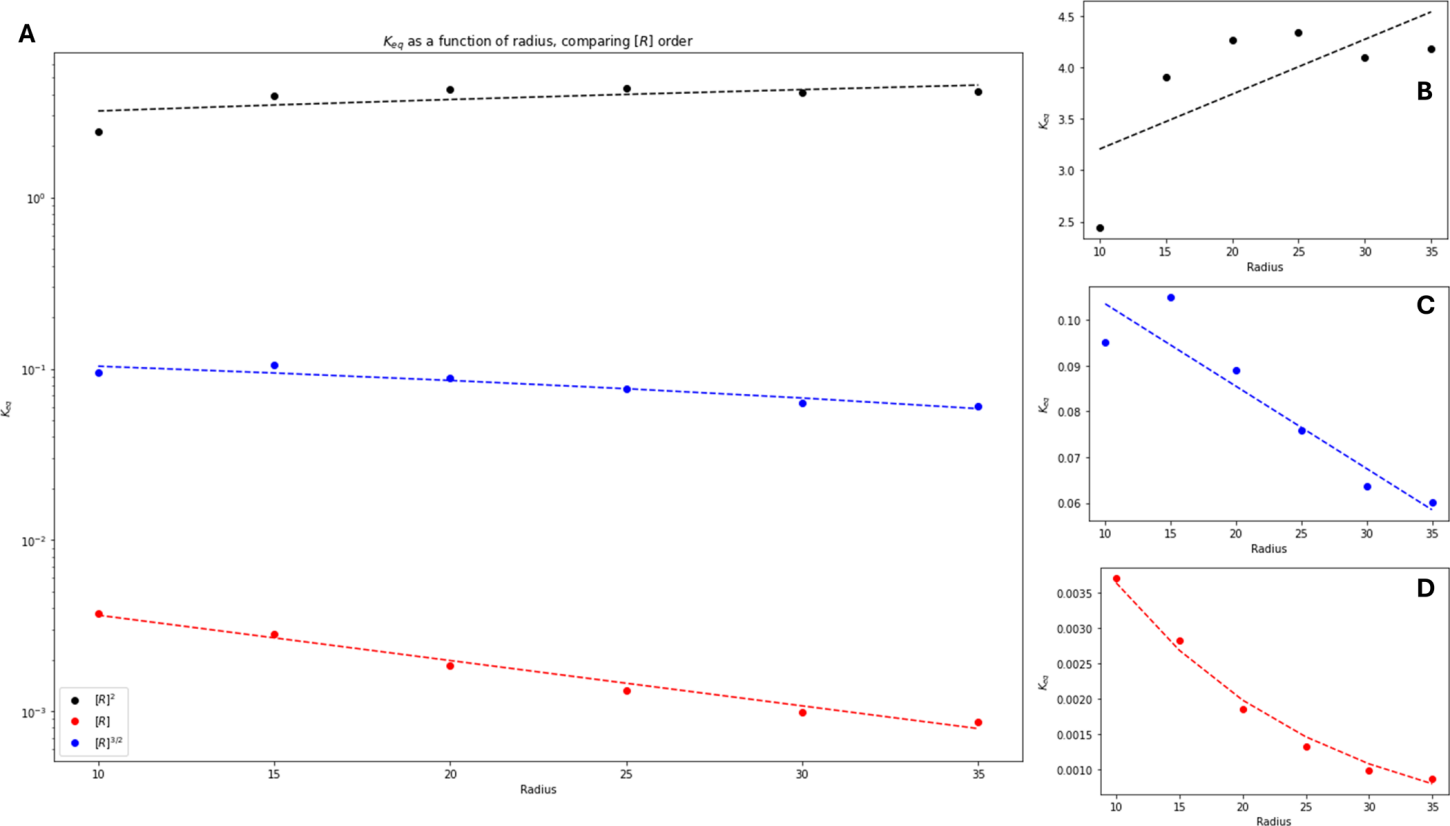
Comparing the effect of radius on various *K*_eq_ calculation schemes. (A) All three schemes of *K*_eq_^*R*^, *K*_eq_^*R* ^3/2^^, *K*_eq_^*R* ^2^^, (B) plots *K*_eq_^*R* ^2^^ as a function of radius, (C) plots *K*_eq_^*R* ^3/2^^ as a function of radius, and (D) plots *K*_eq_^*R*^ as a function of radius.

From these results, *K*_eq_^*R*^ was selected as the reaction *K*_eq_ criteria, as the exponential relationship between *K*_eq_ and the radius, a direct indicator of *M*_0_ and *M*_1_, fits the expected energetic relationship between Minkowski functionals and Gibbs’ free energy described in eq 9. This is because an exponential relationship resolves the left side of eq 9 to a linear form, allowing for the relationship described in Boelens et al. on the right side to hold true.

In examining the unit cell behavior of the benchmark dissolution reaction, a range of radii from 10 to 40% of the unit cell edge length was tested. In terms of *M*_0_, this resulted in a porosity range of 0.5 to 0.95. As shown in Figure 10, an exponential relationship was found between the terms and *K*_eq_^*R*^, with a negative exponential found relating to radius, which corresponds to a positive exponential with respect to *M*_0_. The greatest deviation from this trend in the plot can be found at the largest radius (and thus lowest porosity) of the unit cell. This is likely due to the chemical bearing capacity of the unit cell itself. Without a transport means for chemical species to exit the unit cell, lower porosity unit cells likely experience greater chemical exclusion effects due to the spatial nature of surface CRNs.

**Figure 10:**
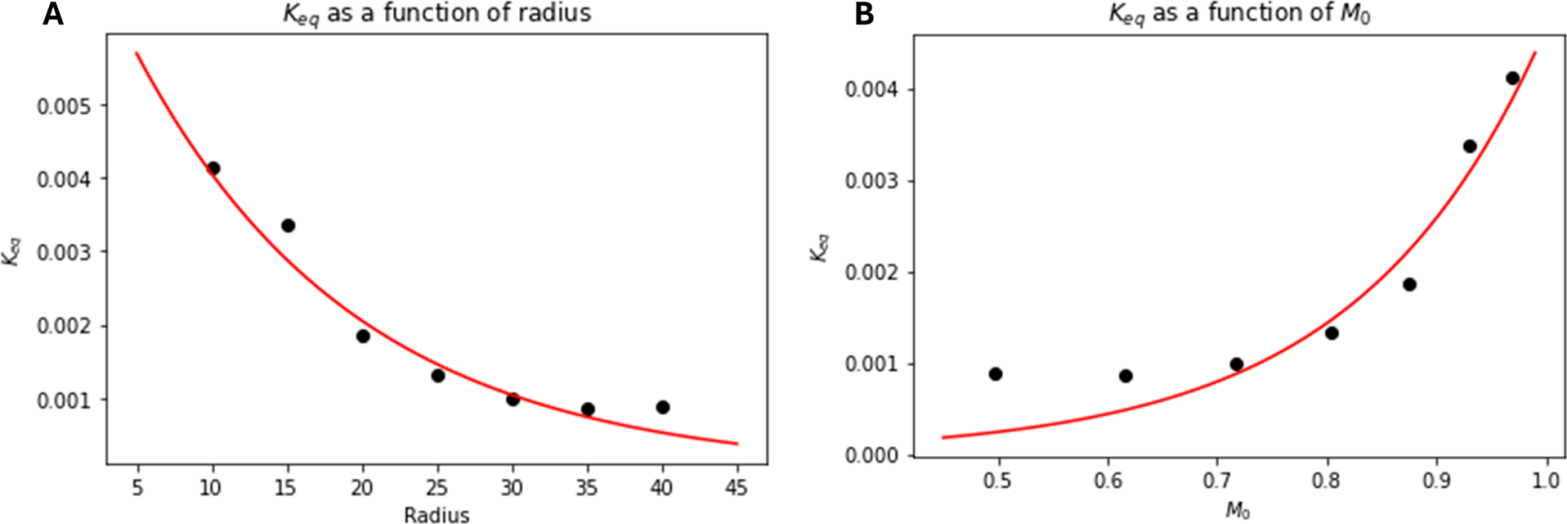
*K*_eq_^*R*^ values for unit cell reactions of a benchmark dissolution reaction. (A) Plots of the evolution of *K*_eq_^*R*^ as a function of unit cell radius. (B) Plots of the evolution of *K*_eq_^*R*^ as a function of *M*_0_.

When plotting the entire *Q* ^*R*^ profile, as seen in Figure 11, the exponential relationship between the radius of the unit cell and the steady state of the system is made clear. There is also a visible relationship between the maximum $\frac{dQ^{R}}{dt}$ and the overall radius of the circles in the unit cell, as seen in Figure 12. This is due to the larger radius of the unit cell providing more potential reaction sites at any given time step, leading to a faster reaction occurring. Notably, while the maximum $\frac{dQ^{R}}{dt}$ varies significantly with radius and porosity, no clear relationship in the time to reach the maximum $\frac{dQ^{R}}{dt}$ , or Δτ, is found, with results visible in the Supporting Information of this text.

**Figure 11:**
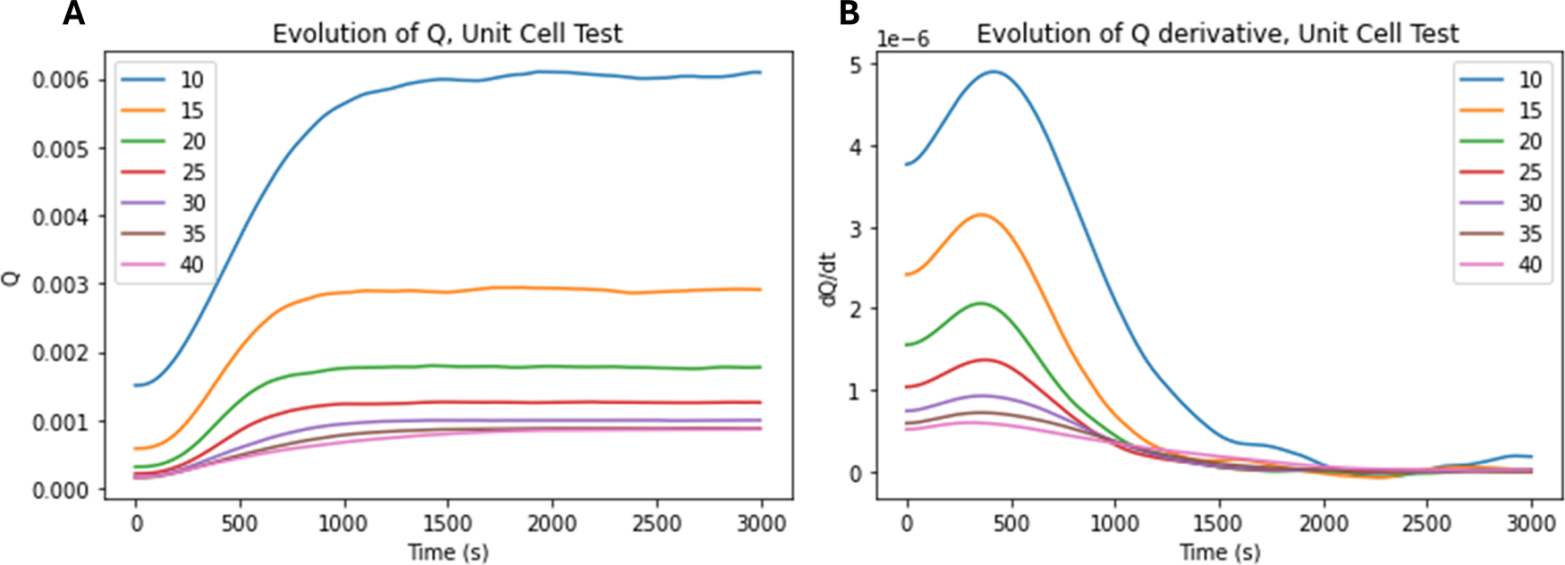
*Q* ^*R*^ values for unit cell reactions of a benchmark dissolution reaction. (A) Plots of the evolution of *Q* ^*R*^ as a function of unit cell radius over time for all test systems. (B) Plots of the evolution of the first derivative of *Q* ^*R*^, $\frac{dQ^{R}}{dt}$ as a function of unit cell radius.

**Figure 12:**
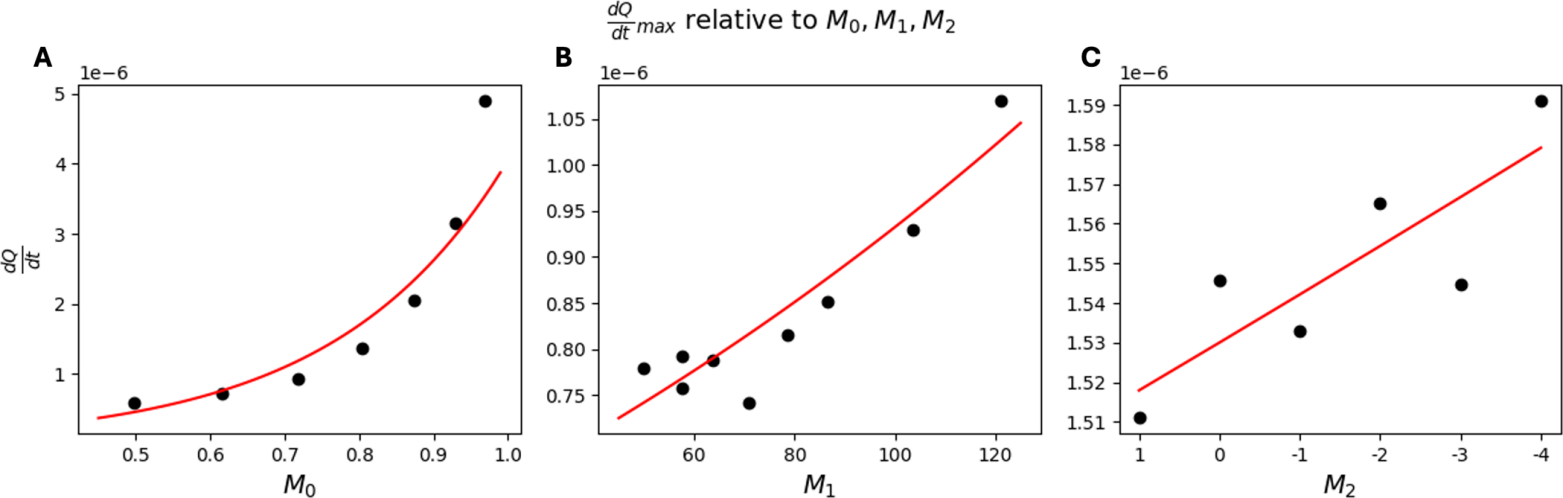
${\frac{dQ^{R}}{dt}}_{\max}$ values for dissolution reaction tests. (A) Plots of the maximum $\frac{dQ^{R}}{dt}$ as a function of *M*_0_. (B) Plots of ${\frac{dQ}{dt}}_{\max}$ as a function of *M*_1_. (C) Plots of ${\frac{dQ}{dt}}_{\max}$ as a function of *M*_2_.

### Perimeter

Perimeter, or *M*_1_, was varied, as described in Section 2 via a 1D diffusive reaction cell. In this reaction cell design, the wave parameters *a* and *b* were varied to generate testing samples with different perimeter value, as seen in Figure 4. Ultimately, these results were combined to draw overall conclusions surrounding the effect of *M*_1_ on the microstructural chemical performance.

From Figure 13, in all cases of perimeter, *K*_eq_ is unchanged outside of minor fluctuations expected of the stochastic nature of Surface CRN experiments. However, in the $\frac{dQ}{dt}$ plots in Figure 13B,D, a clear hierarchy is seen through the relationship of perimeter to $\frac{dQ}{dt}$ behavior visible in the differing slopes of the *Q* ^*R*^ lines. This is further examined in Figure 12, where the relationship between *M*_1_ and the maximum $\frac{dQ}{dt}$ is shown via quantification of the maximum derivative values. Figure 14 demonstrates this relationship in time to maximum $\frac{dQ}{dt}$ (Δτ). In both cases, an exponential relationship is fitted, where the relationship between *M*_1_ and maximum $\frac{dQ}{dt}$ is positive exponential, while the relationship between *M*_1_ and Δτ is negative exponential. This behavior follows the same reasoning as described for the slope of the periodic unit cell, where an increased perimeter leads to more reaction sites, leading to a faster overall reaction. The exponential nature is expected from the log-normal distribution of Minkowski functionals in porous media.^5,1^

**Figure 13:**
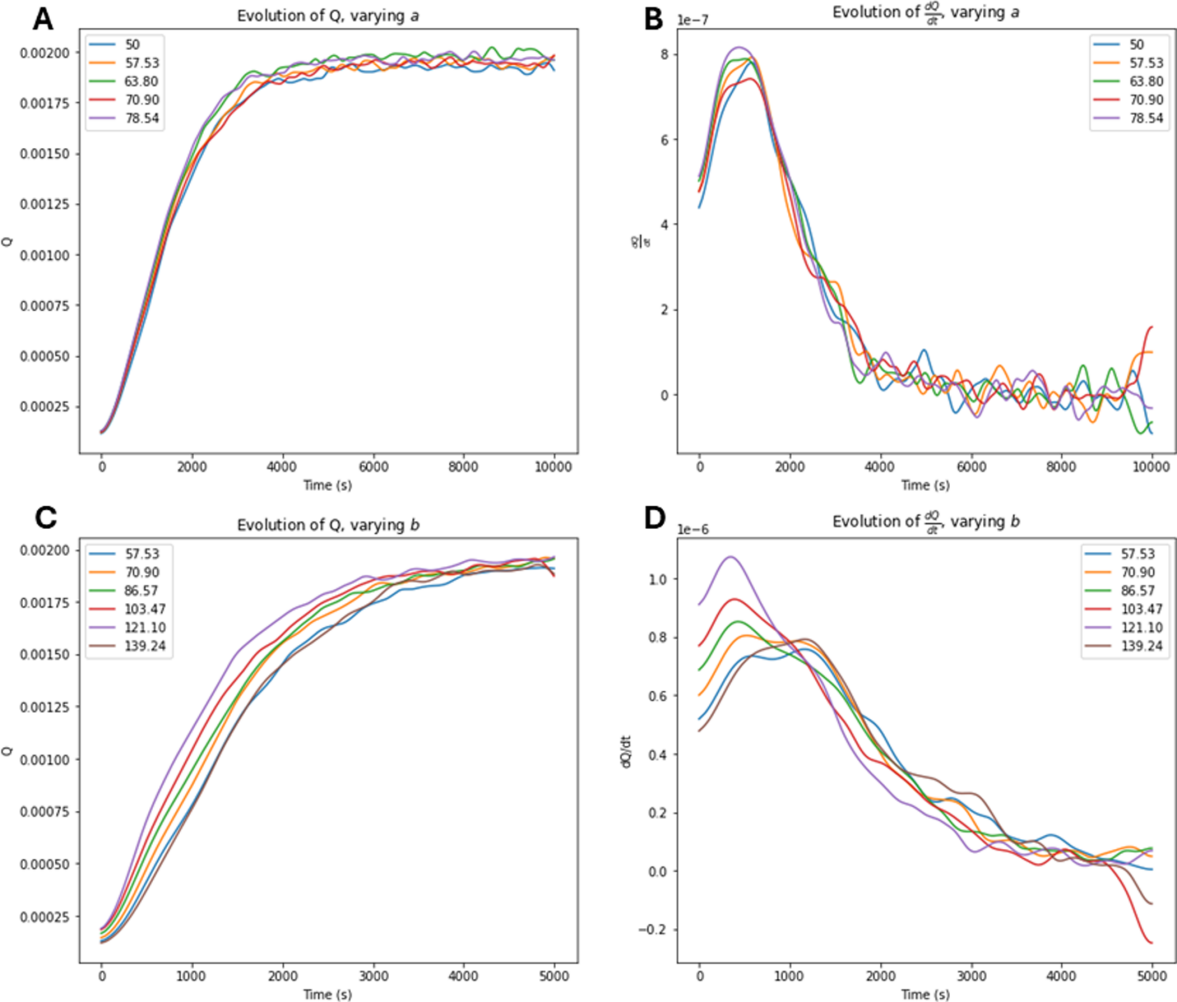
Plots of *Q* ^*R*^ evolution through varying perimeter tests. (A) Overall evolution of *Q* ^*R*^ at different perimeters, controlled by varying *a*. (B) $\frac{dQ}{dt}$ at different perimeters, also through varying *a*. (C) Overall evolution of *Q* ^*R*^ at different perimeters, controlled by varying *b*. (D) $\frac{dQ}{dt}$ at different perimeters, also through varying *b*.

**Figure 14:**
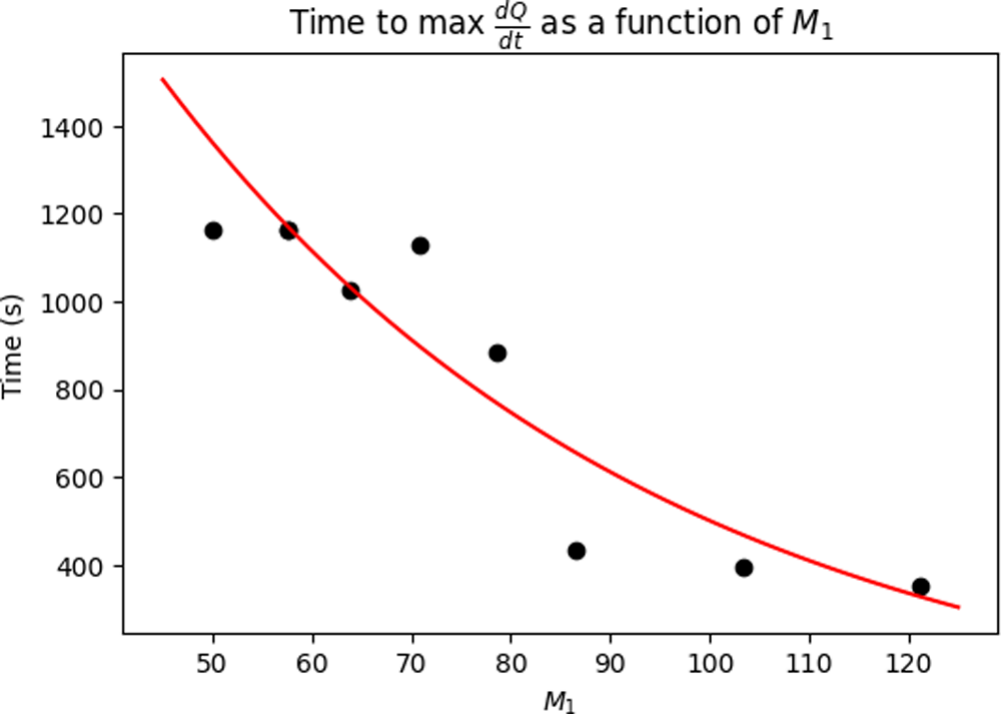
Effect of *M*_1_ on Δτ, demonstrating the exponential relationship between *M*_1_ and the time to maximum $\frac{dQ}{dt}$ .

### Euler Characteristic

As in the preceding sections, the dissolution chemical reaction from eq 2 was applied to the Euler characteristic χ testing scheme described in the methods section. Figure 15A demonstrates that the *K*_eq_^*R*^ of the reaction system seems largely unaffected by the variations of the Euler characteristic. This is further corroborated in Figure 15B, where the overall *Q* profile of each test varies minimally as χ changes.

**Figure 15:**
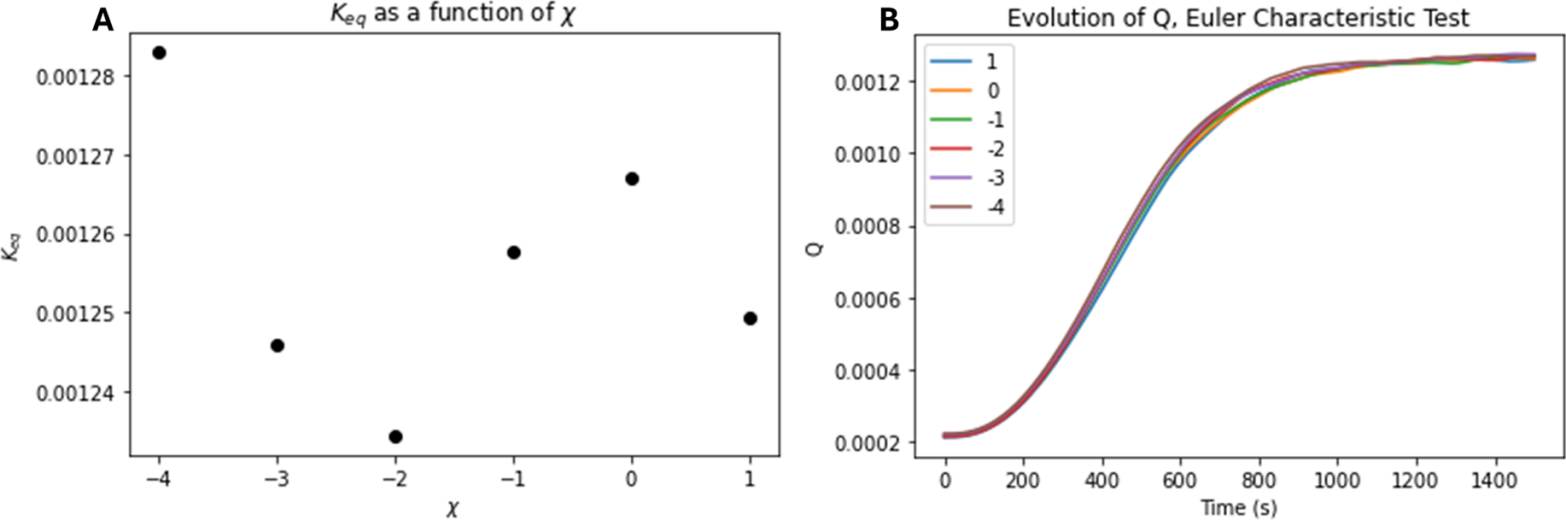
*K*_eq_^*R*^ and *Q* ^*R*^ values for the Euler characteristic χ study of a dissolution reaction. (A) No relationship between *K*_eq_^*R*^ and χ, while (B) corroborates the overall profile of *Q* ^*R*^.

While there is no clear relationship between *K*_eq_^*R*^ and χ, a negative exponential relationship was observed between χ and the maximum $\frac{dQ}{dt}$ as seen in Figure 12, although this is a rather weak relationship in terms of order of magnitude. There was no visible relationship between χ and Δτ.

## Discussion and Conclusions

### Dependency of Reaction to Morphometers

The data extracted from the various unit tests, as shown in Table 2, describes the relationships between the morphometers and chemical reaction properties:

**Table 2:** Reaction Properties and Their Associated Morphometers

	*K*_eq_^*R*^	${\frac{dQ}{dt}}_{\max}$	Δτ
Relevant Morphometers	*M*_0_	*M*_0_, *M*_1_, *M*_2_	*M*_1_

In all cases, exponential relationships were found. Ultimately, the only morphometer with a direct, tangible impact on *K*_eq_ was *M*_0_. This is likely due to the adjustment of *M*_0_ modulating the ratio of reactants available (i.e., a greater *M*_0_ would decrease the amount of reactive solid *A* and increase the amount of reactive fluid ). However, while *M*_1_ and *M*_2_ had a minimal effect on equilibrium behavior, both functionals affected the dynamics of the system– ${\frac{dQ}{dt}}_{\max}$ and Δτ. These effects are likely due to *M*_1_ and *M*_2_ dictating the number of available reaction sites available; that is, the perimeter determines the number of potential interfacial nodes, while χ is a measure of the topological connectivity of the solid phase. In both cases for these dynamic measures, *M*_0_ would be relevant simply for adding more potentially reactive sites to the graph network and more initially reactive species.

### Linkage to Gibbs Free Energy

From the scaling laws linking the Minkowski functionals to the extent of the chemical reaction that we obtained numerically, the following mathematical relationships can be derived. For the equilibrium definition of the standard Gibbs’ free energy Δ*G* ^0^, the following relationship is defined:

$$\Delta G^{0}=-RT\ln\;K_{\mathrm{eq}}$$ 18

When examining the relationships between Minkowski functionals and *K*_eq_, we can define *K*_eq_ as a function of *M*_0_ in the following form:

$$K_{\mathrm{eq}}=K_{0}e^{aM_{0}}$$ 19

for some constants *K*_0_ and *a*, with both being scaling constants linking morphometric relationships derived above to Gibbs free energy linearly. In a similar vein, Δτ can be seen as a function of *M*_1_ in the form:

$$\Delta \tau =\Delta z_{0}e^{bM_{1}}$$ 20

with *b* and *z*_0_ being scaling constants. Finally, this methodology can be applied to ${\frac{dQ}{dt}}_{\max}$ for its relationship with *M* – 0, *M*_1_, and *M*_2_. This takes the form of

$$\begin{array}{rll} {\frac{dQ}{dt}}_{\max} & =Q_{0}(M_{1},M_{2})e^{-cM_{0}} & \\ Q_{0} & =Q_{1}(M_{2})e^{-dM_{1}} & \\ Q_{1} & =Q_{2}e^{-fM_{2}} & \\ {\frac{dQ}{dt}}_{\max} & =Q_{2}e^{-cM_{0}-dM_{1}-fM_{2}} & \end{array}$$ 21

with *c*, *d*, *f*, *Q*_0_, *Q*_1_, and *Q*_2_ being linear scaling constants.

In order to assess the validity of this model, Minkowski functionals for all previous unit cell tests were fed into the model from eq 21, where the predicted $\frac{dQ}{dt_{\max}}$ values were compared against the simulation $\frac{dQ}{dt_{\max}}$ values. Similar additive relationships were also examined, namely, a linear and additive log relationship. These relationships are represented as

$${\frac{dQ}{dt}}_{\max}=Q_{1}+gM_{0}+hM_{1}+jM_{2}$$ 22

and

$${\frac{dQ}{dt}}_{\max}=Q_{1}+k\log(M_{0})+m\log(M_{1})+n\log(M_{2})$$ 23

respectively, with constants *g*, *h*, *j*, *k*, *m*, and *n* representing various linear scaling constants.

From the parity plots in Figure 16, it is clear that not only is the exponential model from eq 21 a substantially more accurate model than that of eqs 22 and 21, its *R* ^2^ value of 0.96 shows that it is quite strong as a predictor on its own. This follows the combination of predicted linear and exponential properties discussed earlier. The values that differed the most, namely at the extreme ranges of $\frac{dQ}{dt_{\max}}$ values correspond with low and high *M*_0_ and *M*_1_ values. These stem from the results of the periodic unit cell test, where low porosity samples begin to overcrowd due to missing the number of nodes needed for the reaction to fully progress, lowering the overall maximum rate of the reaction. A similar situation happens at high porosity, where the rate of diffusion of nodes near future reactive sites becomes a bottleneck for the reaction to proceed forward due to low porosity microstructures having very few adjacent reactive nodes to solids. However, despite these edge cases, the model derived in eq 21 appears to be valid for a thermodynamic relationship derived from Minkowski functionals.

**Figure 16:**
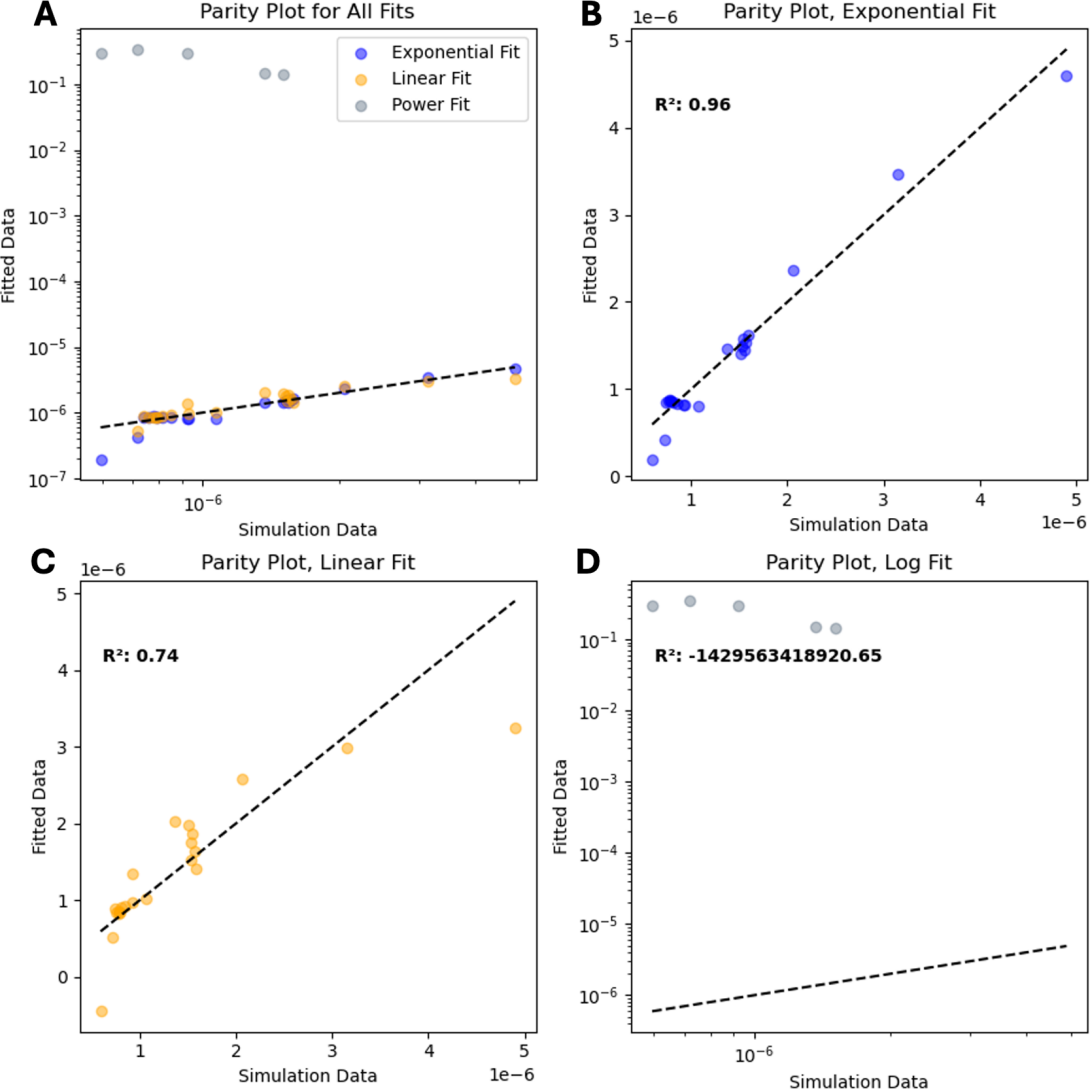
Parity values for the model are described in eq 21. This figure compares simulation results to those predicted from the model and their corresponding *R* ^2^ values. (A) Compares the linear, exponential, and log fits tested. (B) Parity of predicted values to the exponential fit in eq 21. (C) Parity of predicted values to the linear fit in eq 22. (D) Shows the parity of predicted values to the log fit in eq 23.

Minkowski functionals have shown promise in their ability to describe geometrially influenced complex mesoscale phenomena in porous media. Through the use of surface CRNs, a unique model of asynchronous cellular automata, to model dissolution behavior in chemical systems, the effects of Minkowski functionals on the chemical behavior were extracted. Due to the challenges of modeling and characterizing interfacial chemical reactions, the effects of individual simulation hyperparameters were first examined to understand their impact on equilibrium metrics, namely *K*_eq_. Reaction rate scaling showed a simple log–linear relationship in dictating *K*_eq_ behavior, and the dissolution rate appeared to have a direct effect on *K*_eq_. This verifies previous literature that has shown discrepancies in the classical Law of Mass Action and true *K*_eq_ values of nonwell-mixed systems, with these discrepancies related to energetic considerations tied directly to interphase behavior and reaction rates. Beyond the modeled chemistry influence on *K*_eq_ valuation, unique artifacts of the surface CRN simulator must also be taken into account. Specifically, the nature of the reaction selected introduces a branching interface diffusion phenomenon even in models of no assigned chemical diffusion, detailed in Appendix II. This adds an additional layer of slow manifold evolution that must be noted when considering long-term equilibrium behavior. However, as referenced in Appendix II, the fast time scale effects of this diffusion are negligible.

These findings also match the intuition of what is known about dissolution reactions in chemistry, that increasing surface area increases the speed of reaction and increasing the number of reactants in a system decreases the equilibrium constant. While this intuition is well-known in practical applications of chemistry, Minkowski functionals offer a potential quantification for this phenomenon from a geometric perspective.

Ultimately, exponential relationships were found between *K*_eq_, ${\frac{dQ}{dt}}_{\max}$ , and Δτ and extracted Minkowski functionals. With this linkage found and the appropriate scaling quantified, this work stands as an important step in further understanding how Minkowski functionals influence microstructural behavior.

## Supplementary Material



## Figures and Tables

**Figure 17:**
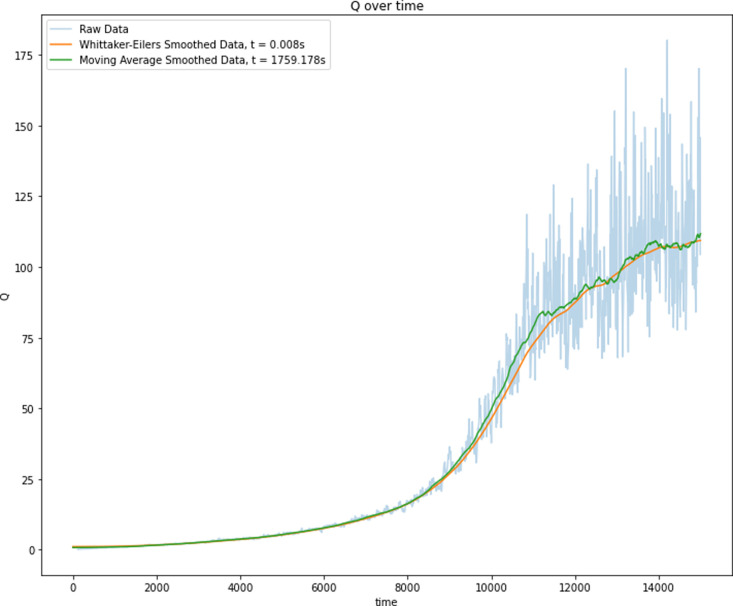
Comparison of filter results on a sample *Q* calculation over 14.7 million data points, comparing a moving average series calculated with a window size of 1,000,000 and a Whittaker–Eilers series with λ = 80,000. Note the time to completion of the smoothing algorithms, with the Whittaker–Eilers smoothing function calculating at a speed 10^6^ times faster.

**Figure 18:**
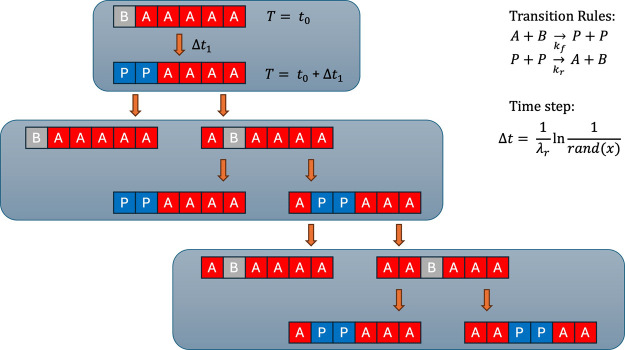
Diagramatic description of branching diffusion.

**Figure 19:**
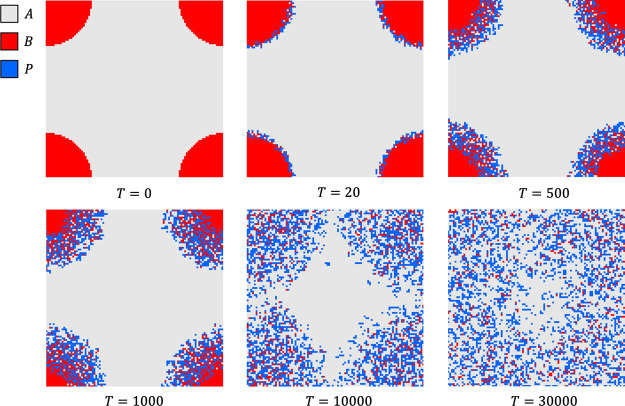
Time evolution of interface diffusion across a periodic unit cell.

## Appendix I: Whittaker–Eilers Filter

Due to the inherent stochasticity of a surface CRN simulation, data generated by these simulations is inevitably noisy, even at a steady state. While the system may have settled into a state of relatively constant concentration, the frequent movement of species due to stochastic switching can lead to small variations in overall species counts. As more species or faster reactions are added to the regime relative to the overall duration of the simulation, the number of concentrations calculated increases dramatically. This compounds the noisy data problem to be incredibly dense, making sensitive calculations of values such as *K*_eq_ and *Q* ^*R*^ messy. In order to properly calculate those global descriptors such as *K*_eq_ and *Q*, chemical data must be smoothed in order to eliminate noise propagation in results. One method for addressing this is through the Whittaker–Eilers smoother, a smoother based on penalized least squares. Extremely fast compared to classic data smoothing techniques like the Savitzky-Golay filter and moving averages, the Whittaker–Eilers filter gives continuous smoothness control as well as automatic interpolation and fast leave-one-out cross-validation. Figure 17 compares the curve generation of the Whittaker-Eilers smoother on noisy *Q* data, showing a marked reduction in data noise similar to that of a moving average calculation, albeit at a fraction of the time to calculate.

Given a set of noisy data *y*, there is a series *z* that is believed to be the optimal smoothness of *y*. As *z* increases in smoothness, the residual between *z* and *y* increases. This residual ϵ is calculated as

$$\epsilon =\sum_{i}(y_{i}-z_{i}{)}^{2}$$
24

and the smoothness *s* of the data is calculated as

$$s=\sum_{i}(z_{i}-z_{i-1}{)}^{2}=\sum_{i}(\Delta z{)}^{2}$$
25

To balance the ϵ and *s* is tuned by the user through the smoothing parameter λ, with the relationship between this quantity represented as *q*:

q=ϵ+λs
26

Ultimately, the Whittaker–Eilers smoother finds the series *z* that minimizes *q*. Combining the above expressions and defining *y* and *z* as vectors **y** and **z** as well as a differential matrix *D*, the expression for *q* evolves to

$$q=\left|y-z{|}^{2}+\lambda \right|Dz{|}^{2}$$
27

Minimizing *q* via setting the gradient of *q* to 0, we arrive at the following expression:

$$\left(I+\lambda D^{T}D\right)z=y$$
28

with **I** defined as the identity matrix. eq 28 is of the form **A** **z** = **y** and can thus be solved via matrix decomposition to find **z**.

## Appendix II: Interface Diffusion Phenomenon as Internal Branching

While the kinetics of the system have direct, tangible effects on the overall behavior of the *K*_eq_ calculation, another important area of consideration is the idiosyncrasies of the simulation medium used in this study. While surface CRNs possess advantages compared to other discrete stochastic simulators, given their inherent spatial sensitivity due to their usage of fixed nodes as well as their simple solving scheme, unexpected secondary behavior may arise depending on the nature of the reaction rules given. In the case of this reaction, a slow but noticeable phenomenon of diffusion was observed to occur, even in models where no diffusion amongst fluids was prescribed.

Typically, in a system where diffusion is disabled (in our case, the rate of the diffusion transition rule is set to 0), chemical reactions occur almost instantaneously at the solid-fluid interface and then stop, creating a layer of product at the boundary. This is because, without some form of transport, reacting species may only form a layer at the surface boundary before the subsequent product shields further reactions from occurring, ultimately terminating the surface CRN simulation early due to the reaction queue collapsing. However, in the case of the class of reaction discussed in this work, the fact that the product of the reversible reaction is two of the same species creates a unique scenario where a slow diffusion manifold is allowed to propagate. As shown in Figure 18, this slow self-propagating diffusion manifold, or internal branching diffusion, is tied directly to the probabilistic nature of asynchronous cellular automata.

After the initiation of a chemical reaction on a surface that generates two identical product species with the contact of a reactive fluid in the presence of a reactive solid *A*, the surface CRN faces a conundrum for its next step: how to resolve the two identical species with the potential for the reverse reaction. As the surface CRN scans each newly generated node species for potential chemical reactions, it finds that both *R* product species are eligible for a subsequent chemical reaction to occur. Thus, both reaction sites draw a random Δ*t* that dictates which of the two sites initiates a reaction first. Depending on which site draws a faster reaction, of which both sites have an equal probability of this occurring, the reverse reaction may assign either site to revert to either or *A*. If this reversibility goes back to the direction of the initial propagation, the reaction oscillates at the boundary between products and reactants. However, if the order of the and *A* reactive sites flips, the dynamics of the reactions change, as now there are sites inside of the solid past the initial boundary that are in contact with reactive nodes. At each step of this flip occurring, new internal potential reaction sites are exposed, propagating the initial reaction through the solid phase. This effect is bidirectional, as these flips may occur in the other direction to move species at the original solid-fluid boundary outward, essentially mirroring a slow diffusion process. With these dynamics incorporated, even with no diffusion prescribed in the transition rules, the ultimate fate of the system at steady state eventually sees the entire solid state dissolve into product, which is dispersed evenly throughout the reacting cell as seen in Figure 19.

While this phenomenon occurs at an incredibly slow rate, with convergence to steady state occurring 30 orders of magnitude in time further than models with even the slowest diffusion constant, this slow manifold directly influences the rate and availability of the reactions in this chemical system and should be noted.

## Notes

Full $\frac{dQ}{dt_{\max}}$ and Δτ results for the unit cell test, varying radius, and thus *M*_0_ and *M*_1_; full $\frac{dQ}{dt_{\max}}$ and Δτ results for the Euler characteristic test (PDF)
