# Supplementary material for: Chemical Homogenization for Nonmixing Reactive Interfaces in Porous Media
Source: ACS Omega. 2025 May 21;10(21):21553–67. doi: 10.1021/acsomega.5c00641 (PMC12138624; doi:10.1021/acsomega.5c00641)
Supplement: Supplementary file 1 [file ao5c00641_si_001.pdf]

Chemical homogenization for non-mixing reactive interfaces in porous media

Winston Lindqwister,[*,†] Manolis Veveakis,[‡] and Martin Lesueur[†]

†*Faculty of Civil Engineering and Geosciences, Delft University of Technology, Stevinweg 1, 2628CN Delft, Netherlands*

‡*Department of Civil and Environmental Engineering, Duke University, 121 Hudson Hall, Campus Box 90287, Durham, NC 27708, USA*

E-mail: w.lindqwister@tudelft.nl

Phone: +31 015 278 9802

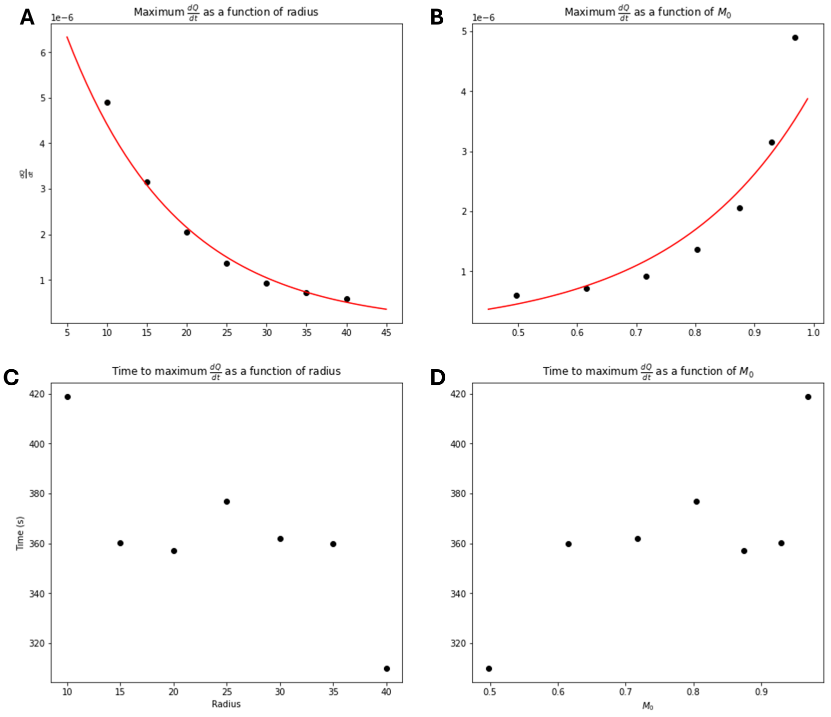

Figure 1: Full $\frac{dQ}{dt_{max}}$ and $\Delta\tau$ results for the unit cell test, both in terms of unit cell radius and M_0–two quantities that are directly related. Both A) and B) show exponential relationships for the evolution of $\frac{dQ}{dt_{max}}$ while C) and D) shows $\Delta\tau$ values with respect to the above variables. As seen in the bottom plots, $\Delta\tau$ stays largely consistent apart from edge cases of the unit cell. This is likely a conflation of the results from the design of the unit cell test with the fact that it does not separate M_0 and M_1. The lower surface area in the higher porosity experiments leads to less available reaction sites compared to the high surface area in the low porosity experiments. However, this is counterbalanced by the fact that high porosity tests have much less reactant in the system, leading to a faster full dissolution of the solid in question. This explains the constant behavior in the middle of the unit cell radius and indicates that at the extremes of the unit cell the influence of the M_1 behavior starts to weigh out the influence of the M0 behavior. This is likely due to the addition of edge case behaviors due to the nature of the simulation—at low porosities, we reach a point where the bearing capacity of the unit cell may be reached due to the size of the unit cell and the number of sites in it. At high porosities, the fact that diffusion must occur at sites bordering the solid before reactions can occur (reaction product P does not react with solid A) means that at very high porosities, reaction products can quickly overcrowd the fewer reaction sites available, leading to an increased time to reach the maximum rate of reaction in the system.

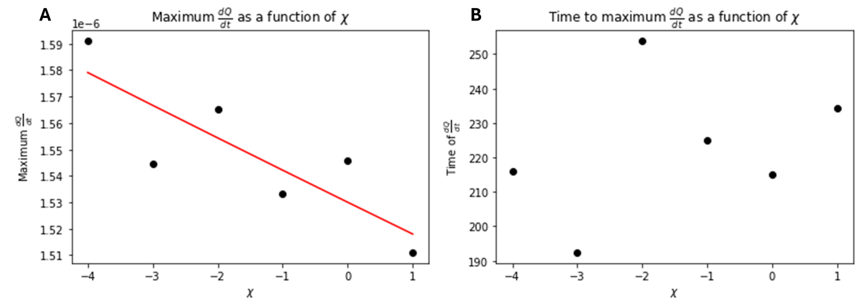

Figure 2: Full $\frac{dQ}{dt_{max}}$ and $\Delta\tau$ results for the Euler characteristic test. A) shows a weak exponential relationship between $\frac{dQ}{dt_{max}}$ and Euler characteristic, while B) shows no relationship between Euler characteristic and $\Delta\tau$.
